# Spontaneous Membrane Nanodomain Formation in the Absence or Presence of the Neurotransmitter Serotonin

**DOI:** 10.3389/fcell.2020.601145

**Published:** 2020-11-30

**Authors:** Anna Bochicchio, Astrid F. Brandner, Oskar Engberg, Daniel Huster, Rainer A. Böckmann

**Affiliations:** ^1^Computational Biology, Department Biology, Friedrich-Alexander University Erlangen-Nürnberg, Erlangen, Germany; ^2^Institute for Medical Physics and Biophysics, University of Leipzig, Leipzig, Germany; ^3^Department of Chemical Sciences, Tata Institute of Fundamental Research, Mumbai, India

**Keywords:** membrane domains, neurotransmitter, serotonin, liquid-disordered domain, molecular dynamics simulation, ordered domains

## Abstract

Detailed knowledge on the formation of biomembrane domains, their structure, composition, and physical characteristics is scarce. Despite its frequently discussed importance in signaling, e.g., in obtaining localized non-homogeneous receptor compositions in the plasma membrane, the nanometer size as well as the dynamic and transient nature of domains impede their experimental characterization. In turn, atomistic molecular dynamics (MD) simulations combine both, high spatial and high temporal resolution. Here, using microsecond atomistic MD simulations, we characterize the spontaneous and unbiased formation of nano-domains in a plasma membrane model containing phosphatidylcholine (POPC), palmitoyl-sphingomyelin (PSM), and cholesterol (Chol) in the presence or absence of the neurotransmitter serotonin at different temperatures. In the ternary mixture, highly ordered and highly disordered domains of similar composition coexist at 303 K. The distinction of domains by lipid acyl chain order gets lost at lower temperatures of 298 and 294 K, suggesting a phase transition at ambient temperature. By comparison of domain ordering and composition, we demonstrate how the domain-specific binding of the neurotransmitter serotonin results in a modified domain lipid composition and a substantial downward shift of the phase transition temperature. Our simulations thus suggest a novel mode of action of neurotransmitters possibly of importance in neuronal signal transmission.

## 1. Introduction

Domain formation in biomembranes is thought to be essential for many biological processes as it may promote the localization of e.g., receptors in a specific sector of the membrane. Thereby, membrane domains facilitate as well the assembly or co-localization of different biological macromolecules by delimiting the accessible two-dimensional space on the membrane surface. Examples include immunological synapse formation (Grakoui et al., 1999; Davis and Dustin, 2004), or GPCR signaling related to dimerization (Gahbauer and Böckmann, 2016, 2020). Membrane domains are defined here as membrane regions confined in space and time, distinguished by membrane composition and/or physico-chemical characteristics from their surroundings (Cebecauer et al., 2018; Kirsch and Böckmann, 2019). These domains have sizes ranging from a few nanometers covering in the order of 10–20 molecules only to more macroscopic domain sizes of a few hundred nanometers (see e.g., Cebecauer et al., 2018). In particular the direct visualization and characterization of small domains is experimentally limited to super-resolution methods such as stimulated emission depletion fluorescence correlation spectroscopy (STED) (Eggeling et al., 2009; Sezgin et al., 2017), interferometric scattering detection (iSCAT) (De Wit et al., 2015; Taylor et al., 2019), or atomic force microscopy (AFM) (Tokumasu et al., 2003; Shan and Wang, 2015). Further experimental evidence for small nanodomains was collected applying Förster resonance energy transfer (FRET) to model membranes (De Almeida et al., 2005; Petruzielo et al., 2013; Koukalová et al., 2017). Other nanoscopic information on membrane domains was achieved using X-ray scattering (SAXS, WAXS) (Sun et al., 2017), neutron scattering (SANS, QENS) (Pencer et al., 2007), or ^2^H NMR (Veatch et al., 2007; Bartels et al., 2008; Bunge et al., 2008; Engberg et al., 2016; Bosse et al., 2019). However, while nanometer spatial resolution could be collected for a number of different model membrane compositions (see Cebecauer et al., 2018 for a comprehensive review), the sub-microsecond and typically as well sub-millisecond dynamics is masked by the limited temporal resolution of the above advanced microscopy techniques, or relies on model assumptions.

The *gap in temporal resolution* was in parts virtually closed—and sometimes also blurred—by the progress in molecular dynamics (MD) simulations during the past two decades. In particular the so-called coarse-grained (CG) simulations characterized by condensation of the degrees of freedom by introduction of super-atoms or super-beads enabled the study of spontaneous domain formation in biomembrane models. The popular MARTINI model provided a first molecular view on domain formation of model membranes with fully saturated and polyunsaturated phospholipids as well as cholesterol, with well distinguished ordered (“raft” domain, or *L*_*O*_) and disordered domains (*L*_*D*_) (Risselada and Marrink, 2008) and has since then been applied in studies on domain formation for various scenarios (Friess et al., 2018; Lin et al., 2018; Bandara et al., 2019). Thereby, frequently little heterogeneity in the composition of individual domains emerged as a characteristics of coarse-grained simulations that is related to the coarsened lipid structure. That is, differences in lipids such as acyl chain length and degree of saturation may at CG resolution easily be exaggerated consequently resulting in pronounced domain separations. In addition, while CG simulations enable for long simulations (micro- to millisecond timescale) coupled to an enhanced dynamics of the membrane constituents (Marrink et al., 2007), the coarsening also affects membrane thermodynamics: The change in degrees of freedom results in a shifted balancing of enthalpy and entropy and thus possibly in a change of temperature-dependency e.g., in domain formation. Nonetheless, CG simulations are in many cases the method of choice to develop a molecular view on domain formation and domain structure.

The experimentally best characterized membrane domains are the sphingomyelin (SM) and sterol-enriched domains that compartmentalize cellular processes (Simons and Ikonen, 1997; Pike, 2006). The size of these domains initially coined *membrane rafts* was given as 10–200 nm (Pike, 2006). More recent experimental studies on model membranes containing phosphatidylcholine (PC), SM, and cholesterol at varying concentrations pointed to domain sizes between approximately 5 and 40 nm (Feigenson and Buboltz, 2001; Pathak and London, 2011, 2015; Koukalová et al., 2017; Saitov et al., 2020). Frequently, these domains were discussed in the context of ordered (*L*_*O*_) domains introduced early for phosphatidylcholine-cholesterol systems (Hjort Ipsen et al., 1987) and taken as model systems for rafts in cellular membranes. However, based on a combination of Monte Carlo simulations with FRET and z-scan FCS experiments, Koukalová et al. (2017) could show that SM and cholesterol driven nanodomains adopt a fluid and disordered *L*_*D*_-like state with large amounts of PC lipids maintaining domain fluidity. These more dynamic domains were suggested as models of nanometer-sized heterogeneities or nanocompartments in biomembranes. A first atomistic-resolution picture of a SM-PC-Chol lipid bilayer from atomistic MD simulations was gained in 2015: A differential interaction pattern of Chol with SM and PC lipids was reported using experiment-derived compositions for *L*_*O*_ and *L*_*D*_ domains (Sodt et al., 2015). The *L*_*O*_-domain was described by a locally hexagonal substructure. Recently, also a *spontaneous* *L*_*O*_:*L*_*D*_ phase separation was reported for DPPC:DOPC:Chol mixtures (Gu et al., 2020), accompanied by a significant partitioning of Chol to both ordered and disordered domains. In this study, the authors employed the Slipids force field (Jämbeck and Lyubartsev, 2012) shown earlier to perform well also close to the phase transition of PC bilayers (Pluhackova et al., 2016). A spontaneous phase separation or domain formation for sphingomyelin-containing PC membranes was, to our knowledge, not yet reported from atomistic simulations. According to Gibbs (1878), the word *phase* is used here synonymously for domains differing in composition or physical state observed for systems in thermodynamical equilibrium.

Membrane phase or domain formation is not only subject to changes in temperature, pressure, external fields, and lipid composition, but as well to (local) exposure to xenobiotics or endogenous molecules. Of particular interest is the interaction of neurotransmitters with membranes. These are released to the synaptic cleft in response to a signal. Their concentration in synaptic vesicles is as high as 270 mM (Bruns et al., 2000), suggesting a concentration of similar order within the synaptic cleft following exocytosis. Binding of serotonin (5-HT) to lipids would result in formation of a 2-dimensional neurotransmitter (NT) reservoir subject to 2D NT diffusion. Membrane binding drastically enlargens the receptor-neurotransmitter encounter rate and probably facilitates NT entry to membrane-buried receptor ligand-binding sites (Postila and Róg, 2020). A low pH and a high calcium level would in turn impede serotonin association within presynaptic vesicles (Mokkila et al., 2017). In a companion paper to this manuscript we describe by means of ^2^H NMR spectroscopy that addition of serotonin to a POPC/PSM/Chol mixture induces increased differences in the lipid acyl chain order between ordered and disordered membrane domains (Engberg et al., 2020). However, detailed knowledge on the interaction with and preference of serotonin (and other neurotransmitters) for plasma membrane (models) displaying *L*_*O*_ and *L*_*D*_ phases is lacking, albeit forming an important cornerstone in the understanding of the role of membranes for neurotransmitter dynamics and possibly linked neurological diseases.

The interaction of the neurotransmitter serotonin with simple phosphocholine lipid bilayers has been described before by means of atomistic MD simulations: Peters et al. (2013) analyzed the interaction of serotonin with single-component 1,2-dipalmitoyl-*sn*-glycero-3-phosphocholine (DPPC) and 1,2-dioleoyl-*sn*-glycero-3-phosphocholine (DOPC) for both charged and neutral forms of serotonin (pK_*a*_ = 9.97 for primary amino group) on the 100 ns timescale. A protonation-dependent binding orientation of serotonin was reported, with the charged amine interacting with the lipid phosphate moiety and the deprotonated serotonin adopting a reversed orientation with the primary amine pointing toward the membrane core. The cationic serotonin form was independently used to investigate the effect of lipid acyl chain unsaturation (Azouzi et al., 2017): Serotonin was shown to preferentially bind to lipids with unsaturation on both chains. Here, however, both the hydroxyl group as well as the charged amino group of serotonin were seen to interact with the interfacial lipid headgroup region, in disagreement with the orientations reported by Peters et al. (2013), independent of the particular protonation state. Another simulation study addressed the binding of different neurotransmitters to model membranes mimicking intra- and extracellular membrane leaflets. The protonated form of serotonin was found to bind to both membrane compositions, albeit at a higher degree to the anionic intracellular membrane mimic (Postila and Róg, 2020). A charged serotonin was as well used in a simulation-based activation study of the 5-HT_3*A*_ serotonin receptor (Guros et al., 2020). Whether serotonin is indeed charged when bound to lipid membranes has to our knowledge not been studied. Also, little is known about differences in interaction patterns of neurotransmitters with ordered or disordered membrane domains, or lipid rafts.

Here, employing atomistic molecular dynamics simulations on the 10 μs timescale, we study the spontaneous formation of ordered and disordered domains for POPC/PSM/Chol mixtures at a molar ratio of 4:4:2 as a model for the extracellular leaflet of plasma membranes in the absence or presence of the neurotransmitter serotonin. The lipid composition was chosen to be in the *L*_*D*_/*L*_*O*_ coexistence region at similar temperatures as studied here, as observed using ^2^H NMR (Bartels et al., 2008; Engberg et al., 2016), EPR (Ionova et al., 2012), and fluorescence measurements (De Almeida et al., 2003; Veatch and Keller, 2005). Concomitant zeta potential measurements on vesicles of the same composition suggest that serotonin stays deprotonated, i.e., uncharged within the membrane. Overall, we observed an enhanced binding of serotonin to the *L*_*D*_ domain resulting in changes in *L*_*D*_ composition, decreased phase transition temperature, and enhanced dissemination of this phase.

## 2. Materials and Methods

### 2.1. Molecular Dynamics Simulations

We conducted 4–10 μs long atomistic simulations of POPC:PSM:cholesterol bilayers at a molar ration of 4:4:2 (mixed *L*_*O*/*D*_ system), 8:61:31 (*L*_*O*_ system), and 69:23:8 (*L*_*D*_ system) in the absence or presence of 10 mol% serotonin (5-HT). The simulations were performed at a temperature of 303 K (*L*_*O*_, *L*_*D*_, *L*_*O*/*D*_) and at 294 and 298 K (*L*_*O*/*D*_). The lipid compositions for the *L*_*O*_ and *L*_*D*_ systems were selected according to a FRET-based phase diagram for a brain SM:DOPC:cholesterol composition using the tie line ends (Enoki et al., 2018) and a corresponding simulation study (Sodt et al., 2015). Similar tie line end compositions for *L*_*D*_ and *L*_*O*_ phases were reported for the ternary POPC:PSM:cholesterol system (De Almeida et al., 2003; Ionova et al., 2012). A summary of the simulated systems is provided in Table 1.

**Table 1:** Simulated systems.

**System**	**Molar**	**Temperature**	**Duration**	**Number of**	**Number of**	**phase**
**composition**	**ratio**	**(K)**	**(μs)**	**lipids**	**5-HT**	
POPC:PSM:Chol	8:61:31	303	4	510	0	*L*_*O*_
POPC:PSM:Chol:5-HT	8:61:31:10	303	6	510	51	*L*_*O*_
POPC:PSM:Chol	69:23:8	303	4	508	0	*L*_*D*_
POPC:PSM:Chol:5-HT	69:23:8:10	303	5	508	51	*L*_*D*_
POPC:PSM:Chol	40:40:20	294	8	510	0	*L*_*O*/*D*_
		298	10	510	0	*L*_*O*/*D*_
		303	10	510	0	*L*_*O*/*D*_
POPC:PSM:Chol:5-HT	40:40:20:10	294	10	510	51	*L*_*O*/*D*_
		298	10	510	51	*L*_*O*/*D*_
		303	10	510	51	*L*_*O*/*D*_

5-HT has two pH-sensitive groups: the primary amino group (NH_2_) and an aromatic hydroxyl moiety (OH) (**Figure 5A**), with pKa values of 9.97 and 10.73, respectively (Peters et al., 2013). Hence, serotonin in aqueous environment at neutral pH is cationic. However, the low dielectric constant environment (ϵ_*r*_ ≈ 2 in the membrane hydrophobic core; Böckmann et al., 2008) inside the membrane could favor the neutral form of 5-HT, as suggested by the measured zeta potential of vesicles in the presence of 5-HT (see below). Since the binding preference to lipid bilayers was shown before for both the charged and the neutral forms of serotonin (Peters et al., 2013), we here modeled only the neutral form of serotonin as the assumed prevalent form of membrane-bound serotonin.

Bilayer systems were initially set up using the CHARMM-GUI web service (Lee et al., 2016). The total number of lipids in each system was between 508 and 510, and the lipids were solvated with at least 38 TIP3P waters per lipid, a salt concentration of 0.15 M NaCl, and an initial box size of ~ 11 × 11 × 9.8 nm^3^. Serotonin molecules were added to the water phase of the systems at random orientation, and at a minimum distance of 2 nm from the lipid headgroups using the *gmx insert-molecules* tool implemented in GROMACS 2019 (Abraham et al., 2015). All the simulations were performed with the GROMACS 2019 software suite (Abraham et al., 2015) together with the CHARMM36 force field (Klauda et al., 2010). Serotonin parameters (Supplementary File 1) were generated with the CHARMM general force field (CGenFF) v4.0 (MacKerell et al., 1998; Vanommeslaeghe et al., 2010, 2012; Vanommeslaeghe and MacKerell, 2012) using the ParamChem web server v2.2.0 (CGenFF, 2020).

Each system was minimized, heated, and equilibrated and then integrated under NPT conditions using a Nosé-Hoover thermostat (Nosé, 1984; Hoover, 1985), and a Parrinello-Rahman barostat (Parrinello and Rahman, 1981) with a compressibility of 4.5 × 10^−5^ bar^−1^. The integration timestep was set to 2 fs, and bonds to hydrogens were constrained with the LINCS algorithm (Hess et al., 1997). Long-range electrostatics were computed using particle-mesh Ewald (Darden et al., 1993) with a tolerance of 10^−6^, 4th-order spline interpolation, and a maximal mesh size of 0.12 nm; van der Waals interactions were shifted smoothly to zero between 1.0 and 1.2 nm.

### 2.2. Data Analysis

Unless noted otherwise, the last 3 μs of each trajectory were used for the analyses, with frames taken every 1 ns. The analyses were performed using custom scripts involving the Scipy (Virtanen et al., 2020), Numpy (Oliphant, 2015), scikit-learn (Pedregosa et al., 2011), and MDAnalysis (Michaud-Agrawal et al., 2011) python packages. The HMM analysis was implemented based on the hmmlearn python library. Visualization and snapshot representation was done with VMD v 1.9.4 (Humphrey et al., 1996).

### 2.3. Analysis Protocol for Lipids Order States and Domains

In the *L*_*O*/*D*_ coexistence systems, the state of the lipids was determined using a hidden Markov model (HMM) analysis, similar to the approach of Park and Im (2019). We decided not to use the local lipid composition as emission signal (Sodt et al., 2014; Gu et al., 2020) but rather the lipid order: Lipid chains and cholesterol orientation time series, quantified through the director order parameter *P*2 (Yankova et al., 2012), were used as observables to define three hidden states: putative *L*_*O*_, *L*_*D*_, and an intermediate ordered state. Briefly, the director order parameter is defined as *P*2 = 1/2〈3*cos*^2^α − 1〉, where α is the angle between the membrane normal and the end-to-end vector of the hydrophobic part of cholesterol or the hydrophobic tail of phospholipids. For phospholipids, |*P*2| was calculated as the average of both tails. The parameters of the model are the probabilities of each hidden state to have a certain *P*2 order, and the probabilities for a hidden state to stay or to change to the other one in a one-time step. The primary assumption is that the probability of the observable emission states, for each lipid type can be decomposed into those from high and lower order states and approximated by a combination of two normal distributions *N*(*P*2|μ_*L*/*O*_, σ_*L*/*O*_), with means μ_*L*_, μ_*O*_ and standard deviations σ_*L*_, σ_*O*_ (Park and Im, 2019). This was possible for all the lipid types in the simulated systems.

As in Park and Im (2019), we trained a simple discrete HMM consisting of nine emission states, assigned by partitioning the observable space into nine subspaces (0,…,8). The lowest and highest emission states are assigned when *P*2 < μ_*L*_ and *P*2 > μ_*O*_, respectively. The intermediate transition states *i*(*i* = 1, …, 7) are assigned when μ_*L*_ + (*i*−1)Δ*P*2 ≤ *P*2 ≤ μ_*L*_ + *iΔP*2, where Δ*P*2 = (μ_*O*_ − μ_*L*_)/7. For the low and high hidden ordered states, the initial emission probability matrix was set up making use of the integrals of the normal distributions over the subspace of the observables. For the intermediate ordered state, we considered integrals over a normal distribution with variance μ_*I*_ = (σ_*L*_μ_*O*_ + σ_*O*_μ_*L*_)/(σ_*O*_ + σ_*L*_), and σ_*L*_ = min[|μ_*O*_ − μ_*I*_|, |μ_*L*_ − μ_*I*_|]/3 to ensure that the intermediate states initial emission probabilities are localized within 3σ between the upper, μ_*O*_, and the lower, μ_*L*_, bounds (Park and Im, 2019). Given these initial set of parameters, the Baum-Welch algorithm (Baum et al., 1970; Rabiner, 1989) was used to find the maximum likelihood estimate of the parameters given as input the time series of the emission states for each lipid type in a given leaflet. Once the HMM parameters are determined, the most likely order state sequence for each lipid was determined by the Viterbi algorithm (Viterbi, 1967).

For better comparison of the hidden ordered, disordered, and intermediate states between simulations with/without serotonin and between simulations at different temperatures (*L*_*O*/*D*_ systems), the emission states and the initial transition and emission probabilities were based on the POPC/PSM/Chol 4:4:2 simulation at 303 K.

Time-weighted histograms were computed to analyze the dynamical properties of the assigned ordered/disordered hidden states to POPC and PSM lipids. This analysis was performed on the last 3 μs of the *L*_*O*/*D*_ system at 303 K with and without serotonin.

### 2.4. Calculation of Deuterium Order Parameters

Deuterium order parameters were calculated at each position along the aliphatic chains of POPC and PSM according to

(1) $$\begin{array}{r} S_{CH}=1/2\langle 3cos^{2}\theta -1\rangle \end{array}$$

where θ is the angle between the C-H bond and the membrane normal (taken to align with *z*, while the bilayer is in the *xy*-plane). Angular brackets denote an average over all sampled configurations. The order parameters were calculated by first averaging over time separately for each lipid in the system and then calculating the average and the standard error of the mean over the different lipids.

The lipids are divided into three populations for the calculation based on the HMM analysis.

### 2.5. Density Probability Distributions

Density probability distributions of water, lipids headgroups glycerol (POPC) and sphingosine backbones (PSM), and serotonin centers of mass along the bilayer normal, *z*, with respect to the bilayer center were computed for the last microsecond using the *gmx density* GROMACS 2019 tool (Abraham et al., 2015). The profiles of serotonin were not symmetrized as unsymmetrized profiles provide a useful check on convergence.

### 2.6. Potential of Mean Force (PMF)

Potential of mean force profiles of serotonin's center of mass were calculated from the last μs of the trajectory according to *F*(*z*) = −*k*_*B*_*Tlnp*(*z*). *p*(*z*) is the probability of finding serotonin at position *z*, and *z* is the position along the bilayer normal with respect to the membrane center, *T* is the absolute temperature, and *k*_*B*_ the Boltzmann constant. The probability density *p*(*z*) was estimated using a Gaussian mixture model (Glodek et al., 2013; Bochicchio et al., 2018).

### 2.7. Serotonin-Bilayer Absorption and Serotonin Orientation

Serotonin absorption and association with the bilayer was determined using its hydroxyl oxygen atom *z*-position along the bilayer normal. The bilayer was divided into a headgroup and a hydrophobic core region based on the density probability distributions of selected groups of the phospholipids and the sphingolipids along the bilayer normal, as described above. Specifically, the headgroup region was assigned as the region between the choline and the glycerols *z*-positional probability distribution, the hydrophobic core as the region corresponding to the hydrocarbon chains.

Serotonin orientation in the bilayer-associated states was analyzed in terms of its indole group orientation. Two angles, θ and α were used to define the indole orientation according to a reference coordinate system depicted in **Figure 5**. The *z*-axis is perpendicular to the indole rings system, bisecting both the benzene and the pyrrole rings. The *x*-axis bisects the plane of the benzene ring, and the *y*-axis is orthogonal to the *x*- and *z*- axes. θ is the angle between the *z*-axis and the bilayer normal, and α defines the angle between the *x*-axis and the projection of the bilayer normal in the *x*-*y* plane. A θ value of 90^o^ corresponds to an indole orientation with its plane on average parallel to the bilayer normal. α values of ~120^o^ degrees and ~310^o^ correspond to an indole orientation in which the hydroxyl oxygen atom is oriented toward the water phase. The percentage of serotonin residing within the hydrophobic core or the headgroup region was determined by calculating the relative number of contacts of serotonin's center of mass and the phosphate groups or the lipid acyl chains, respectively.

### 2.8. Area Compressibility (*K*_*A*_)

The instantaneous area per lipid *A*_*l*_(*t*) for the bilayers was calculated as the area of the simulation cell *A* divided by the number of lipids per leaflet. This assumes that undulations are small so the difference in projected and local areas is negligible. The area compressibility *K*_*A*_ is evaluated from:

(2) $$\begin{array}{r} K_{A}=\frac{k_{B}T\langle A\rangle }{\langle \delta A^{2}\rangle }=A{\left(\frac{d\gamma }{dA}\right)}_{T} \end{array}$$

Where 〈*A*〉 is the average total area, 〈δ*A*^2^〉 is the mean square fluctuation, *k*_*B*_ the Boltzmann constant, and *T* the temperature. The estimation of the standard error was performed using the block average method (Allen and Tildesley, 2017), with blocks of 10 ns (*L*_*D*_) to 150 ns (*L*_*O*_), corresponding to twice the correlation time of the time series fluctuations, estimated from the normalized correlation function of the average total area fluctuations.

### 2.9. Membrane Bending Modulus (*K*_*C*_)

The bilayer bending modulus, *K*_*C*_, was determined using the method developed by Watson et al. (2012), which allows reliable estimates of *K*_*C*_ to be extracted from simulations of modestly sized boxes. This method analyzes the spectral thermal fluctuations of the lipid director vector field $\hat{n}$. The lipid director is a vector pointing from the lipid head to tail and serves as a mean to quantify the lipid orientation. The theoretical prediction for the power spectrum of the longitudinal component of $\hat{n}$ reads:

(3) $$\begin{array}{r} S\left(q\right)=\langle |{\hat{n}}_{q}^{\left|\right|}{|}^{2}\rangle =\frac{k_{B}T}{K_{C}q^{2}}. \end{array}$$

Deviations from Equation (3) are observed only over wavelengths shorter than 3 bilayer thicknesses (Watson et al., 2012). Previous simulations have shown that simulations with 288 lipids only are sufficient to determine *K*_*C*_ (Levine et al., 2014). For an in-depth description of the method, the reader is referred to Watson et al. (2012) and Levine et al. (2014).

### 2.10. Materials

Cholesterol (Chol), 1-palmitoyl-2-oleoyl-sn-glycero-3-phosphocholine (POPC), N-palmitoyl-D-erythro-sphingosylphosphorylcholine (PSM) of highest quality were purchased from Avanti Polar Lipids (Alabaster, AL, USA). 5-HT was purchased from Merck (Darmstadt, Germany). All other chemicals were of highest purity with exception of organic solvents which were of spectroscopic grade.

### 2.11. Lipid Sample Preparation and Zeta Potential Measurements

Lipids were mixed in organic solvents at the molar ratio POPC/PSM/Chol 4/4/2 and evaporated at 40° C in a rotary evaporator. Afterwards the lipids were hydrated at 40° C for 30 min using K_2_PO_4_ buffer (20 mM K_2_PO_4_, 100 mM NaCl, 0.1 mM EGTA pH 7.4 in Milli-Q water), freeze thawed and finally extruded through two 100 nm polycarbonate filters at 40° C to make unilamellar vesicles (LUV). Lipid samples were diluted from a LUV stock solution to a concentration of 0.3 mM for the zeta potential measurements. Concentration of 5-HT in the samples varied between 0 and 10 mM. Serotonin was added externally from a K_2_PO_4_ buffer (12.5 mM 5-HT, 20 mM K_2_PO_4_, 100 mM NaCl, 0.1 mM EGTA pH 7.4 in Milli-Q water). The samples were measured at 25° C after 10 min incubation after externally adding 5-HT. Zeta potential was measured using a Zeta potential analyzer (Brookhaven) with 10 runs and 10 cycles.

## 3. Results

The spontaneous formation of biomembrane domains was addressed in atomistic MD simulations for a POPC/PSM/Chol mixture (4:4:2, *L*_*O*/*D*_ system) in the absence or presence of serotonin at three different temperatures (294, 298, and 303 K, each 8–10 μs). In addition, for comparison of the domain characteristics and quantitative binding analysis of serotonin, MD simulations of previously *in silico* characterized *L*_*O*_ and *L*_*D*_ domain compositions (Sodt et al., 2015) were employed as well. Convergence of the different systems was evaluated by monitoring the (overall) area per lipid and the number of lipid-lipid contacts for the different species (Supplementary Figures 1–3). The bilayer area equilibrated within ≈ 1 μs with a small drift to smaller areas in particular for the *L*_*O*/*D*_ system at low temperature (294 K). Similarly, we observe small drifts in the number of lipid-lipid contacts as a measure of the formation of differently composed domains. Here, we thus focus on the initial steps of domain formation in ternary mixtures containing spingomyelin in the presence or absence of the neurotransmitter serotonin, domain composition, and domain influence on the mechanical membrane properties. The simulation system size and simulation length (10 μs) does not allow to conclude on the formation of large domains beyond 10 nm. Also domain compositions and sizes analyzed below may likely further slowly adapt on the 100 μs to millisecond timescale.

Membrane domain formation was analyzed using a Hidden Markov Model (HMM) employing P2 order parameter-distinguished emission states (see section 2 for details). For an unambigous assignment and for quantitative comparison of systems with/without serotonin as well as for comparison of simulations at different temperatures, the emission states were defined based on the *L*_*O*/*D*_ simulation at 303 K.

### 3.1. Serotonin Charge

As a measure of the charge of serotonin bound to phospholipid membranes we determined the zeta potential for a POPC/PSM/Chol (4:4:2) mixture in the absence or presence of varying concentrations of serotonin. The zeta potential (see Table 2) was hardly affected even for high serotonin concentrations and varied between −0.1 and −4.5 mV, suggesting that serotonin preferentially binds in an uncharged i.e., deprotonatated form to the membrane. Also for pure POPC membranes no effect of serotonin on the zeta potential could be seen (data not shown). Accordingly, serotonin was chosen uncharged in all simulations.

**Table 2:** Zeta potential of a POPC/PSM/Chol 4/4/2 membrane for different serotonin concentrations.

**Serotonin concentration**	**0 mM**	**0.1 mM**	**0.25 mM**	**0.5 mM**	**1 mM**	**5 mM**	**10 mM**
Zeta potential (mV)	−4.48	−1.39	−2.46	−4.2	−2.13	−0.09	−0.71
Std. Error	2.43	2.62	2.02	0.58	1.03	1.24	2.25
Ser:lipid molar ratio	0	0.33	0.83	1.67	3.33	16.67	33.33

### 3.2. Spontaneous Nanodomain Formation in Sphingomyelin-Rich PC Membranes

**Figure 1:**
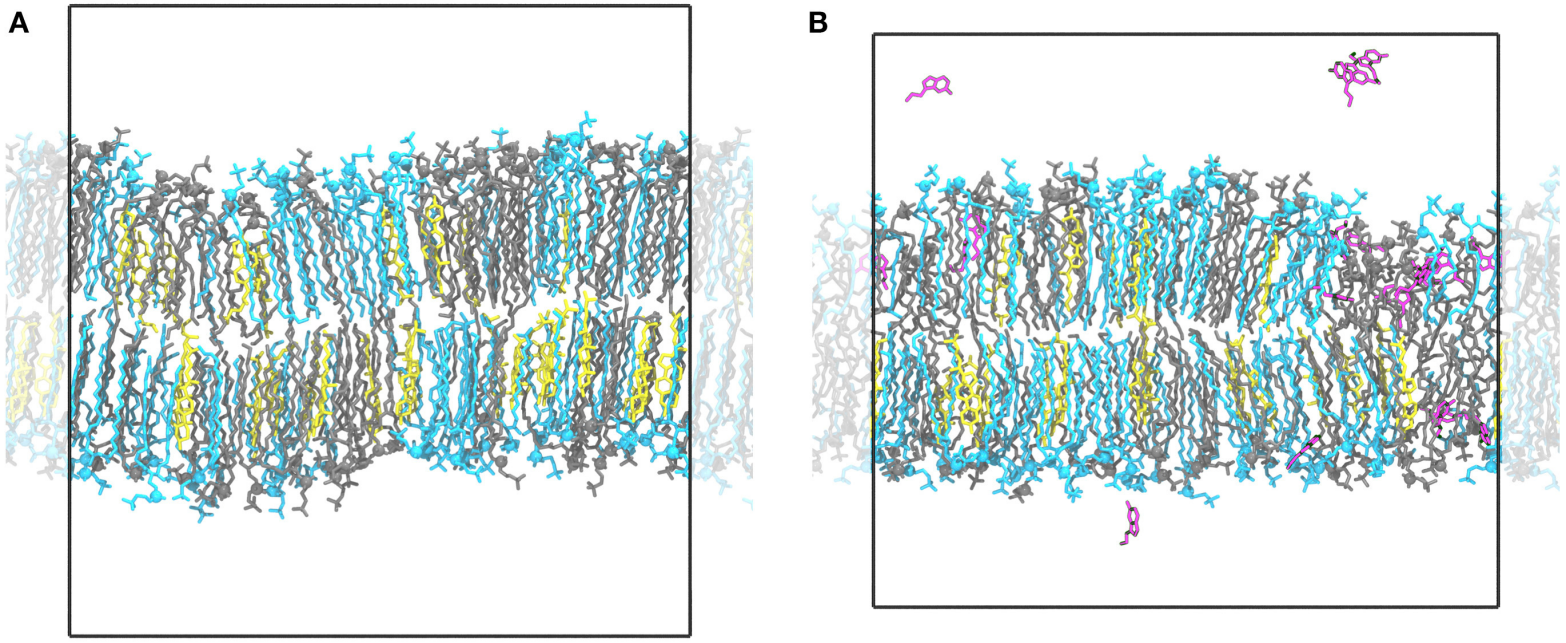
Ordered and disordered membrane domains. Shown are snapshots of the *L*_*d*_/*L*_*o*_ membrane (POPC:PSM:Chol at 4:4:2) at 294 K without **(A)** and with serotonin **(B)**. In the absence of serotonin, the membrane is overall highly ordered with ≈ 60% of all lipids assigned to the ordered HMM state. In the presence of serotonin, larger disordered and diluted, serotonin-enriched lipid domains emerge suggesting a serotonin-induced phase transition. POPC lipids are depicted as *blue* sticks, PSM lipids in *gray*, cholesterol molecules in *yellow*, and serotonin molecules in *magenta*. Water molecules are omitted for clarity.

**Figure 2:**
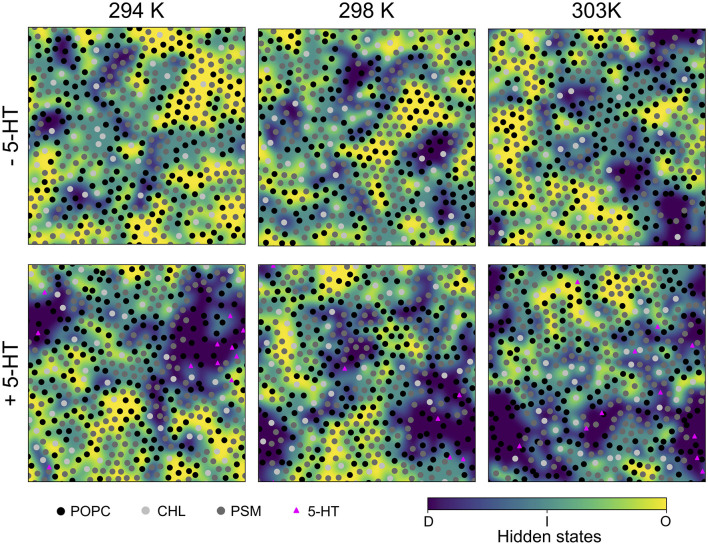
Last snapshots of HMM-assigned lipid order states. A Hidden Markov Model was used to assign ordered (“O,” *yellow*), disordered (“D,” *violet*), and intermediate (“I,” *light blue*) states to lipids based on their P2 order parameters as emission states. The emission states and initial probabilities were derived from the *L*_*O*/*D*_ system at 303 K. The lipid center of mass positions are marked by shaded circles, the positions of bound serotonin by triangles.

Membrane domain formation was monitored for the *L*_*O*/*D*_ simulation (POPC/PSM/Chol 4:4:2 mixture). The HMM analysis revealed spontaneously formed ordered, sphingomyelin-enriched domains (referred to as *L*_*O*_ domain, see Figure 1 for snapshots) that include between 48% of all lipids (303 K) and 60% (294 K, compare Table 3). At 303 K, the *L*_*O*_ domains contained ≈ 43% PSM, 34% POPC, and 23% Chol while the composition of the more disordered *L*_*D*_ domain changed hardly with respect to the overall 4:4:2 composition (see Table 3). Interestingly, the *L*_*I*_ domain (including lipids in intermediate ordered state) is observed to be enriched in POPC (52%). That is, the sphingomyelin-enriched *L*_*O*_ domains are surrounded by PC-enriched intermediate regions that transition into a more mixed POPC/PSM *L*_*D*_ domain. That indeed coexisting domains are formed is clearly discernible from representative snapshots displayed in Figure 2 (see also corresponding movies in the Supplementary Material): In particular at 303 K, larger regions of disordered domains formed that display a more loose lipid packing. In contrast, the POPC/PSM/Chol system at 294 K is mostly found in its ordered state (i.e., *yellow*) with only small more disordered patches, however at a comparably high lipid packing density. These differences in the formation of loosely packed *L*_*D*_ domains are as well reflected in the average area per lipid. At 294 K, the area per lipid is ≈ 53 Å^2^ and increases to ≈ 56.5 Å^2^ at 303 K (Supplementary Figure 1, area per lipid excluding cholesterol). The observed spontaneous formation of domains differing in composition and physical characteristics suggests that the domains approximate corresponding coexistent phases in thermodynamic equilibrium.

**Table 3:** Phase distribution for Chol, POPC, and PSM lipids in the *L*_*O*/*D*_ system. Standard errors are given in parenthesis.

			**303 K**			**298 K**			**294 K**		
**System**		**Lipid**	***% L*_*O*_**	***% L*_*D*_**	**%Int**	***% L*_*O*_**	***% L*_*D*_**	**%Int**	***% L*_*O*_**	***% L*_*D*_**	**%Int**
*L*_*O*/*D*_	w/o 5-HT	POPC	34(2)	39(4)	52(4)	40(2)	30(4)	49(4)	41(2)	28(5)	47(4)
	+ 5-HT		30(2)	46(3)	49(3)	37(2)	38(3)	49(4)	39(2)	37(3)	47(4)
	w/o 5-HT	PSM	43(2)	42(3)	32(4)	40(2)	47(4)	34(4)	41(2)	44(4)	33(4)
	+ 5-HT		43(2)	40(3)	33(4)	41(2)	43(3)	33(4)	41(2)	42(3)	33(4)
	w/o 5-HT	Chol	23(2)	19(3)	16(3)	21(2)	22(3)	17(3)	18(1)	28(4)	19(4)
	+ 5-HT		26(2)	14(2)	18(3)	22(2)	19(3)	18(3)	20(2)	20(3)	20(4)
Domain distribution in %			48(4)	26(3)	25(2)	53(4)	23(3)	24(2)	60(5)	18(4)	22(2)
		+ 5-HT	42(3)	36(3)	22(2)	46(4)	30(3)	26(2)	53(3)	26(2)	21(2)

In the presence of serotonin, the phase characteristics between *L*_*D*_ and *L*_*O*_ differed more significantly. Even at 294 K, a larger disordered domain is formed, lipids in disordered state now account for 26% of all lipids (18% w/o serotonin). Also, the domain composition changed upon addition of serotonin, in particular a significant change in the PC content is observed: While the PC and PSM content in the absence of serotonin is similar for the *L*_*D*_ domain, addition of serotonin led to a significant enrichment of PC within the disordered domain at all investigated temperatures (e.g., from 39 to 48% at 303 K, Table 3). These phase changes are coupled to preferential binding of 5-HT to *L*_*D*_-phase membrane domains (*triangles* in Figure 2, lower panel) as discussed in more detail below.

To further assess the dynamical domain properties we analyzed the lifetime of POPC and PSM lipids within the ordered and disordered hidden states for the *L*_*O*/*D*_ system at 303 K with and without serotonin. In the absence of serotonin, POPC lipid lifetimes showed two clearly distinct populations for ordered and disordered state lipids (Supplementary Figure 4A). The time-weighted distributions were fitted by a double exponential and yield lifetimes of 17 and 51 ns for lipids in the disordered state as compared to 32 and 118 ns for lipids in the ordered state (fits performed on initial 300 ns). Addition of serotonin resulted in a significant stabilization of lipids within the disordered state, the lifetimes for the *L*_*D*_ lipids increased to 20 and 99 ns (Supplementary Figure 4B). Note, however, that the lifetimes are expected to change for longer simulation times with increasing domain sizes. This effect is clearly seen in the number of disordered state lipids with lifetimes beyond 300 ns that is substantially increased in the presence of serotonin with increasing *L*_*D*_ membrane domain sizes. In contrast, the lifetimes of PSM lipids in disordered and ordered states were not significantly affected by addition of serotonin on the short timescale (Supplementary Figure 5). Similar to POPC, the number of PSM lipids that were stabilized within the disordered state increased. These results are in line with the observed preferential binding of serotonin molecules to the *L*_*D*_ phase of membranes (see below).

### 3.3. Phase-Dependent Lipid Order

**Figure 3:**
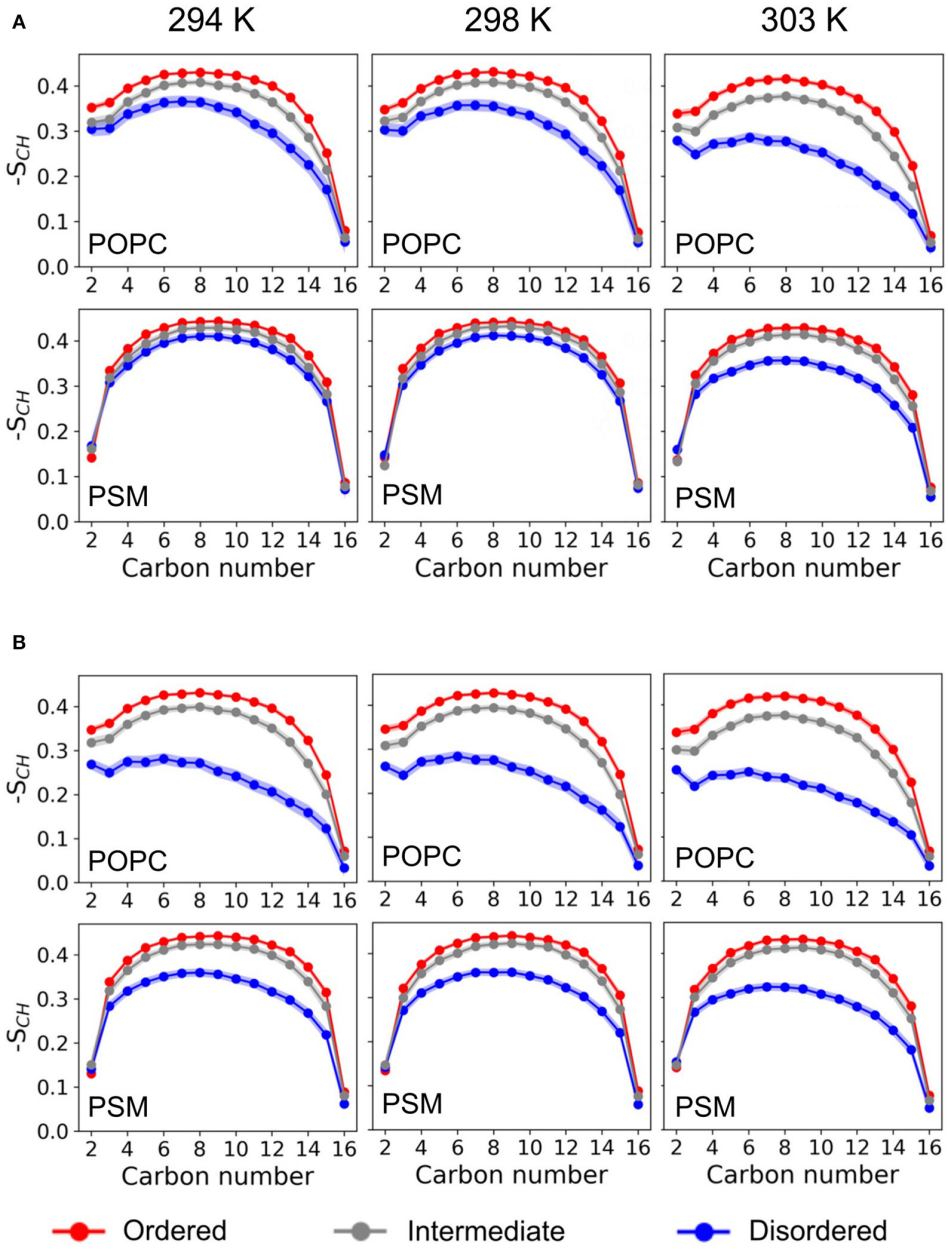
Deuterium order parameter profiles. Shown are the deuterium order parameter profiles for the palmitoyl-chains of POPC (it sn-1) and PSM in the *L*_*O*/*D*_ system at different temperatures for the different HMM-assigned membrane phases (ordered, disordered, intermediate) and without **(A)** or with **(B)** serotonin.

**Figure 4:**
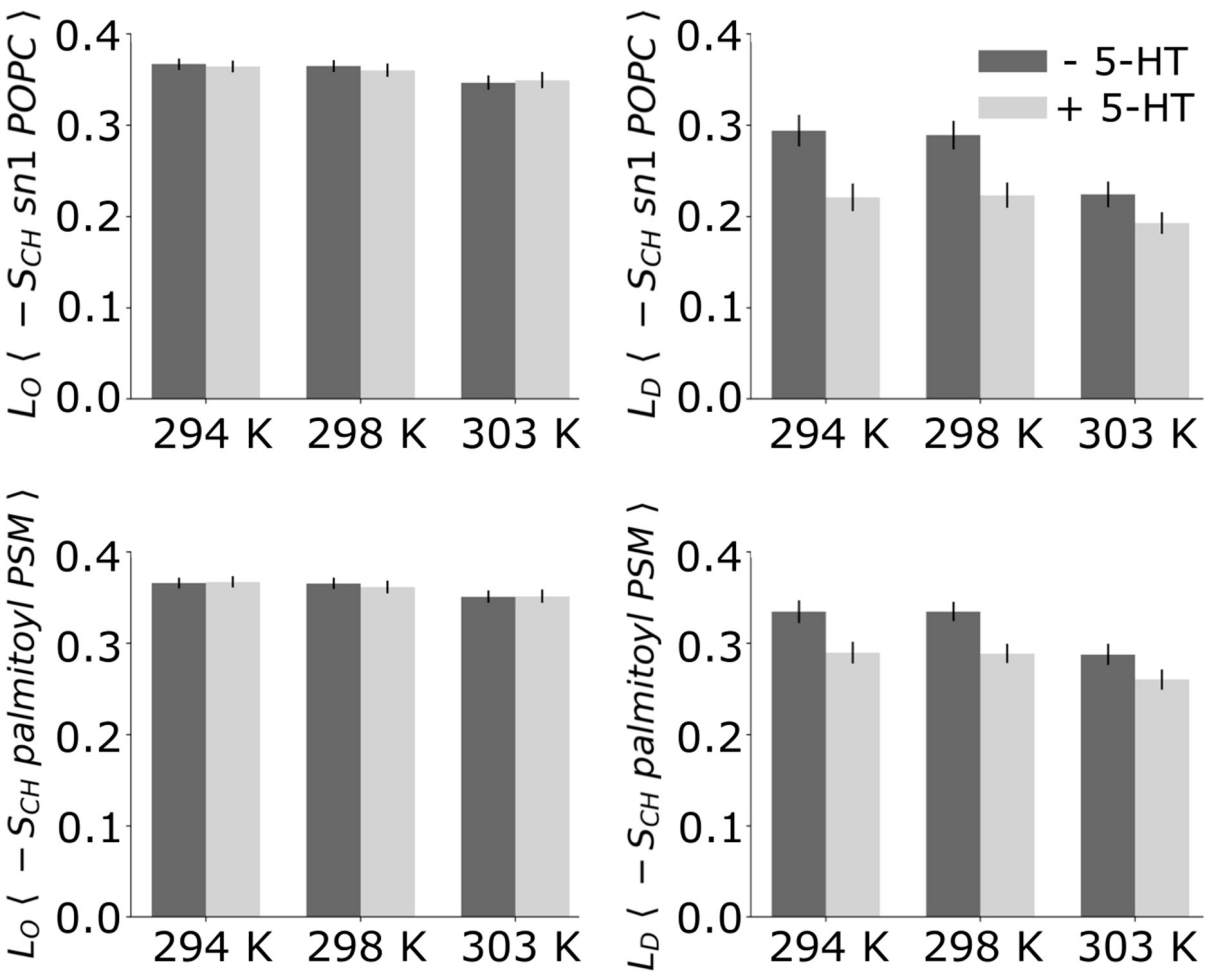
Average deuterium order parameters. Given are the deuterium order parameters averaged over the palmitoyl chain of POPC **(Top)** and the palmitoyl chain of PSM **(Bottom)** without (*dark bars*) and with serotonin (*light bars*).

The lipid acyl chain order provides information on the degree of ordering in ordered and disordered membrane domains. The deuterium order parameters for *L*_*O*_ and *L*_*D*_ phases as determined from the HMM differed substantially for the POPC/PSM/Chol (4:4:2) lipid membranes at 303 K, the average POPC *sn-1*-chain order 〈|*S*_*CH*_|〉 being 0.347 ± 0.008 for the *L*_*O*_ phase and 0.224 ± 0.014 for the *L*_*D*_ phase (see Figures 3, 4). The difference in lipid order diminishes for decreasing temperatures, the *L*_*D*_ *sn-1*-chain order increases to 0.294 ± 0.017 at 294 K while the *L*_*O*_ *sn-1*-order stays approximately constant (0.367 ± 0.006). The comparably high order for lipids assigned a disordered state by the HMM at low temperature is related to the small *L*_*D*_ domain size and hence increased packing density of the lipids (compare Figure 2). That is, lipids in more disordered state are embedded in an overall highly ordered environment. A comparison to ^2^H NMR at 303 K shows a substantially increased POPC *sn-1* order for the *L*_*O*/*D*_ system in the simulations (Engberg et al., 2020). This discrepancy is probably related to an overall shift of the transition temperature to larger values observed for different atomistic force fields (Pluhackova et al., 2016).

The order parameters within the differently ordered domains of the mixed system at 303 K thereby agree well with the palmitoyl chain order determined for pure *L*_*D*_ and *L*_*O*_ systems with compositions equaling those of a previous simulation study (Sodt et al., 2014). Related to the different composition of these 1-phase systems—with POPC:PSM:Chol composition in *L*_*O*_ at 8:61:31 and *L*_*D*_ at 69:23:8—the difference between the average deuterium order parameters for *L*_*O*_ and *L*_*D*_ of POPC is comparable to the *L*_*O*/*D*_ system with 〈|_*S*_*CH*_|〉*O*_ = 0.376 ± 0.009 and 〈|_*S*_*CH*_|〉*D*_ = 0.255 ± 0.042.

Upon addition of serotonin, a strong decrease of the lipid acyl chain order is observed for the disordered domains (for both POPC and PSM) at *all* studied temperatures. In contrast, the order within the *L*_*O*_ domains was hardly affected by serotonin (see Figure 4). While in the absence of serotonin, truly disordered domains are observed only above 298 K, addition of serotonin results in large disordered and loosely packed domains even at 294 K. That is, the serotonin-lipid interaction partially disrupts the lipid packing. The increased disorder of *L*_*D*_ domains as well as their increased size also affect the lipid area: At 303 K, the average lipid area is increased by ≈ 5% with respect to the serotonin-free case (Supplementary Figure 1).

Our results thus suggest a significant serotonin-induced decrease in the phase transition temperature for the POPC/PSM/Chol mixture.

### 3.4. Serotonin-Lipid Interaction

**Figure 5:**
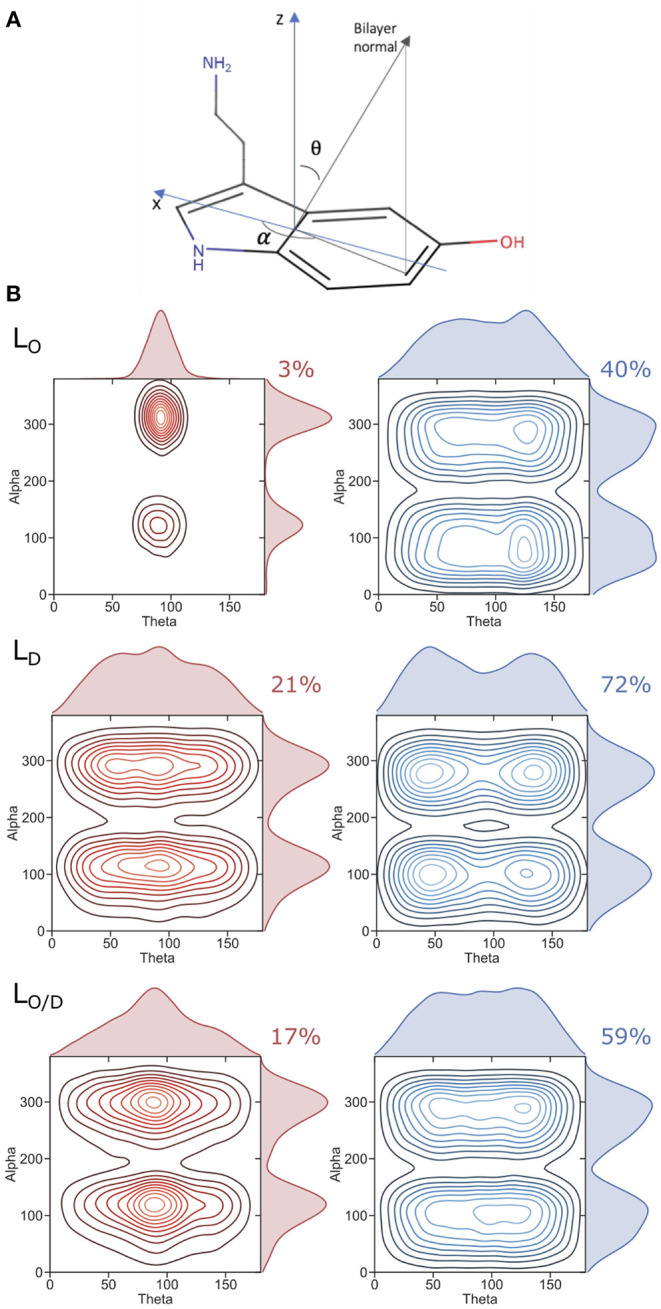
Serotonin orientation in the bilayer. **(A)** Axis framework used to define the orientation of serotonin. **(B)** Plots showing the population of observed angles used to describe the orientation of serotonin for the respective phases (*L*_*O*_, *L*_*D*_, *L*_*O*/*D*_). Red plots (left) correspond to serotonin molecules residing in the hydrophobic core of the bilayer, whereas blue plots (right) correspond to those located within the lipid headgroup region. The percentages of serotonin residing at the hydrophobic core of the bilayer or within the headgroups is also reported.

At simulation start, serotonin was added to the solvent phase of each system at random positions at a lipid:serotonin ratio of approximately 10:1 (in total 51 serotonin molecules, see Table 1). Spontaneous binding of serotonin to lipids was distinguished into binding to either the hydrophobic core region or to the polar lipid headgroup region. For the pre-assembled *L*_*O*_ phase (POPC:PSM:Chol at a concentration of 8:61:31) binding almost exclusively occurred to the lipid headgroup region (Figure 5). In turn, 21 and 17% of all serotonin molecules bound to the hydrophobic core region of membranes with predefined *L*_*D*_ composition or the *L*_*O*/*D*_ membrane, respectively. Also within the *L*_*O*/*D*_ membrane, we observed preferential binding of serotonin to the disordered membrane domains (compare Figure 1 and Figure 2).

**Figure 6:**
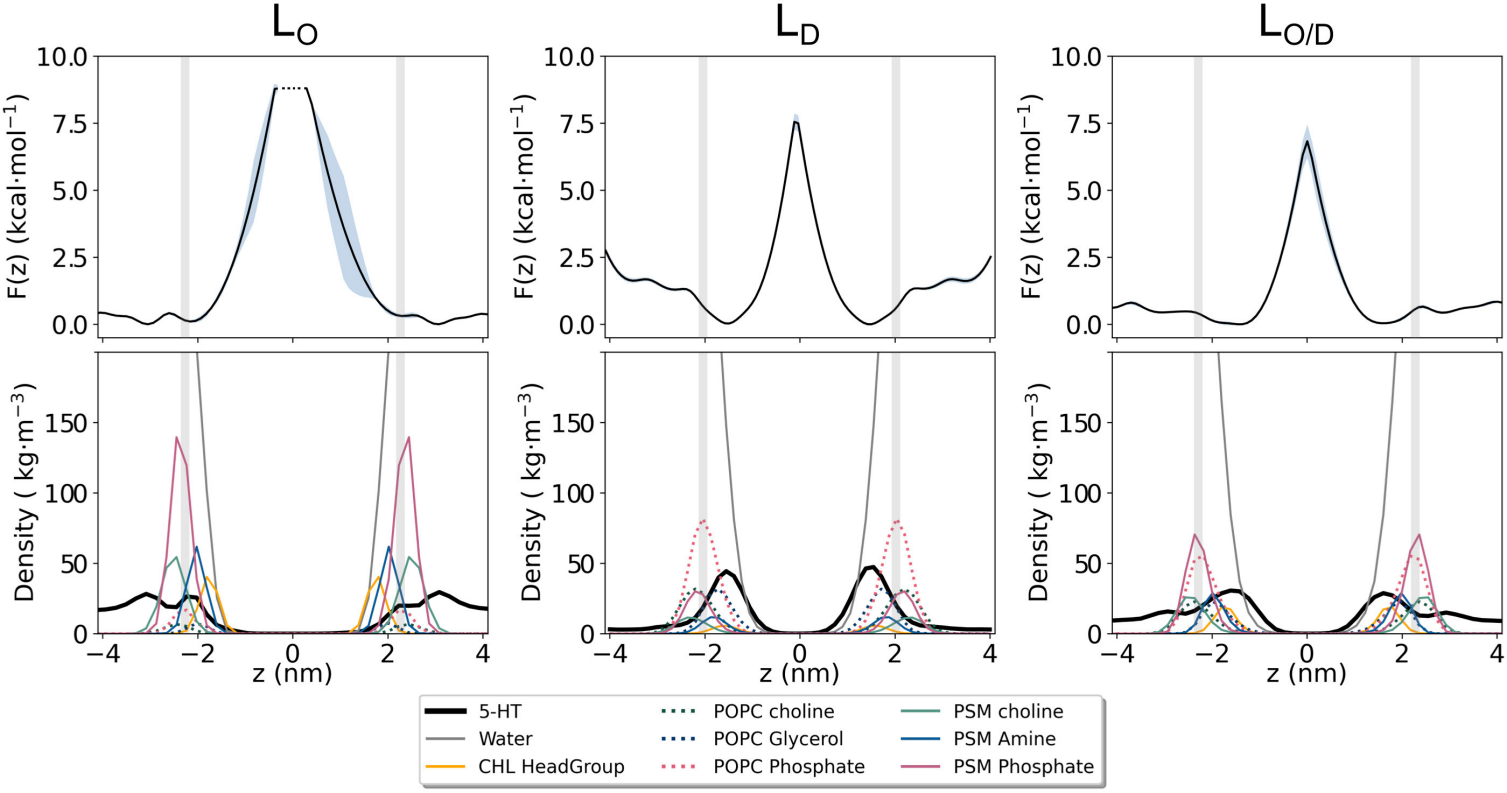
Potential of Mean Force (PMF) for serotonin binding to lipid membranes. Shown is the potential of mean force **(Top)** as estimated from the serotonin density along the membrane normal *z* for the *L*_*O*_, *L*_*D*_, and *L*_*O*/*D*_ phase membranes **(Bottom)**.

The different adsorption modalities of serotonin concerning the membrane phases are well described by the position probability distribution profiles of its center of mass compared to that of selected lipid and cholesterol segments, and water, as well as by the potential of mean force (PMF) profiles. The profiles are shown in Figure 6 and the selected lipids groups are reported therein. The profiles for the phospholipid atoms and water molecules resemble the features that are generally observed in PC-rich bilayers, i.e., broad phosphate and choline distribution profiles, and penetration of water molecules into the lipid headgroup region including cholesterol hydroxyl groups. All the profiles clearly show that serotonin is able to penetrate the bilayer, irrespective of the membrane phase. For the *L*_*O*_-system, the highest probability for 5-HT is within the lipid headgroup region, that is, between the choline group and the glycerol/amine backbones. More than 50% of the serotonin remains in the solvent phase, only ≈ 3% are able to reach the carbon chains. An interesting density drop at the interface between water and the headgroup region is observed. It corresponds to a small but significant free energy barrier in the PMF profile (Figure 6) at the position of the choline group of PSM. In the PC-rich *L*_*D*_ system, serotonin penetrates on average deeper into the membrane. For the *L*_*O*/*D*_ phase the profile is broadened. The same tendency is indicated by the PMF data as well.

The generally broad 5-HT profiles reflect the mobility of the solute in the membranes. An analysis of the orientation of serotonin bound to the membranes is reported in Figure 5. We characterize the orientational distribution of 5-HT by attaching a local coordinate frame (Figure 5) to its indole group. The bilayer is symmetric under rotations about the bilayer normal, so two angles suffice to characterize the indole distribution: θ, the angle between the *z* axis of the indole and the bilayer normal; and α, the angle that is assumed when the bilayer normal is projected into the molecular *x*-*y* plane (compare section 2). Figure 5 shows the probability density of the indoles along these two axes. When bound to the headgroup region, serotonin shows broad orientational distributions for all the considered systems (*right panel*). However, orientations with θ = 50^o^ or θ = 135^o^ are preferred. This can be related to a tendency to form cation-pi stacking interactions with the choline headgroups, experimentally observed for indoles binding to PC lipids (Gaede et al., 2005).

The 5-HT orientation becomes restricted only when it reaches the glycerol backbone and enters the bilayer hydrophobic core (Figure 5, *left panel*). The orientation is dominated by structures with θ near 90^o^—indicating that the molecular plane of the indole is orthogonal to the plane of the bilayer. This is particularly the case for serotonin binding to the *L*_*O*_ system, while a much broader profile is observed for the *L*_*D*_ and the mixed *L*_*O*/*D*_ systems (see Figure 5). The same preferred orientation for the neutral form of serotonin was already observed by Peters et al. (2013) in a previous MD simulation study employing POPC, DOPC, and DPPC membranes. These maxima occur at α values of 120 and 300^o^. In the former orientation, the hydroxyl group is oriented toward the water phase, and the aliphatic amino group points to the center of the bilayer. In the latter orientation, the molecule is rotated by 180^o^ corresponding to serotonin binding to the lower membrane leaflet (with reference to the coordinate system in Figure 5A). That is, for 5-HT embedded within the *L*_*O*_ domains, the carbon chain aligns to the lipid acyl chains.

These results, together with the huge PMF barrier of >7 kcal/mol in the membrane's interior (Figure 6), suggest a very small permeability for this solute, despite high partitioning into the membrane.

### 3.5. Membranes Mechanical Properties

The bending modulus *K*_*C*_ is arguably the single most important quantity in membrane biophysics (Watson et al., 2012) and determines the membrane's ability to resist bending in a number of biologically relevant processes such as membrane fusion (Liu et al., 2006), membrane trafficking of molecules (McMahon and Gallop, 2005), and endocytosis (Chernomordik et al., 1995). As such, larger values of *K*_*C*_ correspond to greater rigidity of the bilayers. The area compressibility modulus, *K*_*A*_, quantifies the response of the membrane area to tension, which under physiological conditions may arise from various perturbations including the addition of small molecules to the membrane.

To our knowledge, no experimental data—area compressibility and bending modulus—are available for the lipid bilayer compositions studied in this work. Hence, a direct comparison of our results to experiment is difficult. However, for the *L*_*D*_ system (see Table 4) the average area per lipid (*APL*) is in good agreement with a previous simulation for the same composition (Sodt et al., 2015), however well below the APL reported for pure POPC bilayers in experiment (Kučerka et al., 2011) and simulations (Pluhackova et al., 2016). This decreased APL arises due to the condensing effect in particular of cholesterol. In addition, the area compressibility modulus, *K*_*A*_, and the bending rigidity, *K*_*C*_, are in line with previous studies of pure PC bilayers at 303 K, reporting *K*_*A*_ = 180−330 mN/m (Binder and Gawrisch, 2001), and $K_{C}=9-13\times 10^{-20}$ J (Binder and Gawrisch, 2001).

**Table 4:** Averaged physical properties of the membrane in the different systems: bending constant (*K*_*C*_ [10^−20^*J*]), area per lipid (*APL* [^2^]), and lipid area compressibility (*K*_*A*_ [mN/m]). Standard errors are given in parenthesis.

		**303 K**			**298 K**			**294 K**		
**System**		***K*_*C*_**	**APL**	***K*_*A*_**	***K*_*C*_**	**APL**	***K*_*A*_**	***K*_*C*_**	**APL**	***K*_*A*_**
*L*_*O*/*D*_	w/o 5-HT	13.0 (0.3)	45.1 (0.1)	552 (3)	10.4 (0.4)	42.8 (0.1)	705 (3)	12.2 (0.4)	42.2 (0.1)	1274 (1)
	+ 5-HT	10.6 (0.4)	47.5 (0.1)	417 (2)	9.2 (0.3)	45.4 (0.1)	543 (2)	9.1 (0.3)	44.9 (0.1)	529 (5)
*L*_*O*_	w/o 5-HT	24.6 (0.8)	40.4 (0.1)	2143 (2)						
	+ 5-HT	23.3 (0.7)	40.5 (0.1)	2140 (3)						
*L*_*D*_	w/o 5-HT	13.4 (0.4)	55.3 (0.1)	266 (0.7)						
	+ 5-HT	11.2 (0.4)	57.8 (0.1)	222 (0.4)						

Extremely large *K*_*A*_ values for mixtures of PSM-Chol (1,718 mN/m) with respect to those of POPC-PSM mixtures (781 mN/m) have been experimentally reported by Needham and Nunn (1990), and related to an additional effect of PSM to decrease area fluctuations, possibly related to the tendency of SM to form intermolecular hydrogen bonds with cholesterol (Henriksen et al., 2004). In line with this observation, we observed a similarly large area compressibility of *K*_*A*_ ≈ 2,140 mN/m for the PSM-rich *L*_*O*_ system (61% PSM, 8% Chol). The average area per lipid for this system is also in full agreement with that reported for the same composition by Sodt et al. (2015). Our values for the bending rigidity of the *L*_*D*_ system of $K_{C}\approx 13.4\times 10^{-20}$ J are in good agreement with experimental values for PC systems (Nagle, 2017). As the experimental values vary and computational reports on Chol and PSM effect on *K*_*C*_ values in PC membranes are lacking, a more quantitative validation for this and the other systems is hampered.

Serotonin absorption and binding to the membrane has a strong effect on all the calculated physical observables. The data show a 16%, and 12−18% decrease in the bending modulus *K*_*C*_ for the *L*_*D*_ system and *L*_*O*/*D*_ systems upon serotonin binding, respectively, as reported in Table 4. Such an effect, together with an increase in the average area per lipid and a strong decrease in *K*_*A*_, indicate substantial membrane softening upon neurotransmitter binding. Little or no change is observed for the pure *L*_*O*_ phase (Table 4), as expected from the observed low serotonin absorption.

## 4. Discussion

Plasma membranes are rich in phosphatidylcholines, cholesterol, and sphingomyelin. In this study, we provide an atomistic view on membrane domain formation of a ternary POPC/PSM/Chol lipid bilayer as a model for plasma membranes. Employing atomistic MD simulations, we trace the initial steps of the formation of (nano-)domains differing in composition, structure, and ordering i.e., dynamics, and mechanics, starting from a random mixture of the membrane constituents. Additionally, we demonstrate that a high neurotransmitter concentration may heavily affect the equilibrium between disordered and ordered lipid membrane domains by preferential binding to *L*_*D*_-domains.

In the ternary mixture, domains characterized by a substantial difference in lipid acyl chain order are formed only above 298 K. Interestingly, and despite the excellent agreement of these different acyl chain orders with those of simulations of systems with predefined compositions for ordered and disordered domains (see also Sodt et al., 2015), changes in domain composition are less distinct on the 10 μs timescale. That is, the ratios of PSM or POPC do not significantly differ between the differently ordered domains. This finding that composition lags behind ordering is most likely partly related to the limited size of the domains formed on the accessible timescale. It is important to note that ordered lipid domains (*L*_*O*_) were defined in literature as domains containing lipids with extended and ordered acyl chains similar to the gel phase or *L*_β_-phase, and at the same time display a high mobility more similar to the disordered *L*_*D*_-phase (Brown and London, 1998; Polozov and Gawrisch, 2006). This rather counterintuitive characteristics, high order combined with high mobility, was supported by pulsed field NMR spectroscopy revealing a lipid diffusion coefficient that differs by only a factor of approximately 3–10 between ordered and disordered phases (Filippov et al., 2004; Orädd et al., 2005; Scheidt et al., 2005). Oppositely, experimental studies showed a comparable fluidity of ordered and gel phases using fluorescence probes (M'Baye et al., 2008). While the simulations yield a large acyl chain order, the lipid diffusion differs substantially between the ordered and disordered phases: The POPC diffusion coefficient within the *L*_*D*_-phase is 4.1·10^−8^*cm*^2^/*s* and is lowered by a factor of ≈ 25 within the ordered phase (diffusion coefficients analyzed for the predefined *L*_*O*_- and *L*_*D*_-phases, see also Pluhackova et al., 2016). Possibly, the different timescales between NMR (several ms) and MD (nanoseconds) impede the comparison. We speculate that, due to the long timescale, NMR may partly average the dynamics of lipids exchanging between ordered and disordered phases and thereby yield an efficient enhanced diffusion.

Most simulation studies dealing with serotonin assumed a protonated amine group of serotonin in agreement with the reported pK_*a*_ of 9.97. However, binding of the neurotransmitter to lipid membranes with a low dielectric constant ( decreasing from ≈ 78 in the solvent to ϵ_*r*_ ≈ 2 within the membrane hydrophobic core; Böckmann et al., 2008) as well as altered hydration properties may substantially shift the pK_*a*_ (Narzi et al., 2008; Sandoval et al., 2016). A recent experimental study addressing the association of serotonin to model lipid bilayers revealed a nonspecific binding of the neurotransmitter at physiologically relevant concentrations (Josey et al., 2020). In the same work, neutron reflectometry results favored a model with deep penetration of serotonin into the bilayer, with its long axis oriented along the membrane normal. A protonated serotonin is at variance with the latter configuration since either the polar hydroxyl group or the charged amine would need to protrude into the hydrocarbon core region of the membrane. This indirect hint to a deprotonated membrane-bound form of serotonin is strongly supported by our zeta potential measurements on lipid vesicles displaying a largely serotonin-independent potential. Accordingly, our simulation study on the influence of serotonin on membrane domain formation focused on the deprotonated i.e., uncharged form of serotonin.

Addition of the neurotransmitter led to a significant downward shift of the phase transition temperature, thereby resulting in clearly distinguished ordered and disordered phases now even at 294 K. This phase shift is induced by preferential binding of serotonin to the domains containing mostly lipids in disordered state, enhancing thereby the fluidity of these domains. This is in agreement with a shift of the main phase transition temperature to lower temperatures reported before for DMPC upon addition of serotonin (Seeger et al., 2007) and similarly for ethanol (Griepernau et al., 2007). However, in the former study the shift induced by 3 mM serotonin was below 1 K. The *L*_*D*_ domains under the influence of the bound neurotransmitters are characterized by an enhanced ratio of phosphatidylcholines, an increased area per lipid, as well as a drastic reduction in the bending modulus and the area compressibility. The latter findings are in apparent contradiction to a companion manuscript (Engberg et al., 2020) reporting in particular ^2^H NMR and AFM indentation experiments on the same ternary biomembrane models. In this work, an increased intendation force was required to rupture the membrane in presence of serotonin and interpreted as an increase in membrane stiffness. These opposing findings, a decreased bending modulus and area compressibility on the nano-scale and an increased membrane stiffness on the AFM scale, may be reconciled assuming that serotonin induces the formation of larger domains as supported by our ^2^H NMR experiments (Engberg et al., 2020). Additionally, serotonin induces a more pronounced disorder of the *L*_*D*_ domains (this work and ^2^H NMR; Engberg et al., 2020) as well as a more pronounced order of the *L*_*O*_ domains (^2^H NMR). The latter may be explained by changed domain compositions as observed in the simulations displaying an increased PC content of the *L*_*D*_ domains and a thus likely decreased PC content for (larger) ordered domains and thereby increased ordering. Interestingly, in good agreement with our simulation data, a recent work reports a significant softening of lipid bilayers containing POPC/POPG/Chol (1:1:1) by serotonin (Dey et al., 2020).

In summary, the initial steps of the spontaneous lateral segregation of a plasma membrane model containing POPC/PSM/Chol (4:4:2) is observed on the microsecond timescale. Despite small differences in compositions, formed nano-domains display a huge difference in order resembling those for PC-rich disordered and PSM/Chol-enriched raft-like ordered domains. The neurotransmitter serotonin reshapes in particular the disordered membrane domains resulting in a PC-enrichment and a decreased membrane elasticity and probably in increased membrane domain sizes as suggested by comparison to ^2^H NMR (Engberg et al., 2020). These changes in membrane structure, domain formation and domain size, and membrane dynamics will probably among others affect receptor sorting and oligomerization in plasma membranes (Gahbauer and Böckmann, 2020), as well as membrane stability, and related permeability and pore formation (Kirsch and Böckmann, 2016, 2019). A possibly similar mechanism was recently revealed for inhalation anesthetics that were shown to increase the size of ganglioside (GM1) rafts and to change the localization of the phospholipase D2 to GM1-enriched domains (Pavel et al., 2020).

Regarding its action as neurotransmitter, our results suggest a fast binding of serotonin to the postsynaptic membrane following synaptic vesicle fusion and thereby a fast and efficient mechanism for neurotransmitter removal from the synaptic cleft (Postila et al., 2016). The preferential binding of serotonin to *L*_*D*_-domains with a concomitant change in composition, ordering, and reduced phase transition temperature likely as well alters the membrane lateral pressure profile as reported before also for ethanol (Griepernau and Böckmann, 2008). A changed membrane pressure may in turn be hypothesized to shift the equilibrium between activated/non-activated receptor configurations if assuming different cross-sectional areas for different receptor activation states (Cantor, 1997a,b, 1998). In addition, high concentrations of membrane-bound neurotransmitters would result in efficient binding to membrane-buried receptor binding sites.

## Data Availability Statement

The raw data supporting the conclusions of this article will be made available by the authors, without undue reservation.

## Author Contributions

RB designed the research. ABo setup and carried out the simulations. ABo and ABr analyzed the simulations. OE performed the zeta potential measurements and analysis. RB wrote the paper with contributions from all coauthors. All authors contributed to the article and approved the submitted version.

## Conflict of Interest

The authors declare that the research was conducted in the absence of any commercial or financial relationships that could be construed as a potential conflict of interest.
