# Supplementary material for: Spontaneous Membrane Nanodomain Formation in the Absence or Presence of the Neurotransmitter Serotonin
Source: Front Cell Dev Biol. 2020 Nov 30;8:601145. doi: 10.3389/fcell.2020.601145 (PMC7734319; doi:10.3389/fcell.2020.601145)
Supplement: Supplementary file 3 [file Data_Sheet_1.PDF]

Supplementary Material

1 SUPPLEMENTARY DATA

Serotonin parameters are provided as GROMACS topology files separately.
Supplementary movies are submitted separately for the $L_{O/D}$ system with and without serotonin at 303 K.

1.1 Computation of contacts

Contacts between each lipid type were computed between the headgroup atom of each lipid (P, for POPC and PSM and O3 for Chol). The cut-off for each pair was chosen based on its radial distribution function coming from the $L_{O/D}$ system at 303 K. The cut-off for all interactions with Chol was fixed to 8.55 Å, based on the first minimum between Chol-Chol, whereas the cut-offs for the other pairs corresponded to the second minimum of their radial distribution function. This was chosen since the first two minima are close to each other and by choosing the second one we allow for alternative orientations in the headgroups.

2 SUPPLEMENTARY TABLES AND FIGURES

2.1 Figures

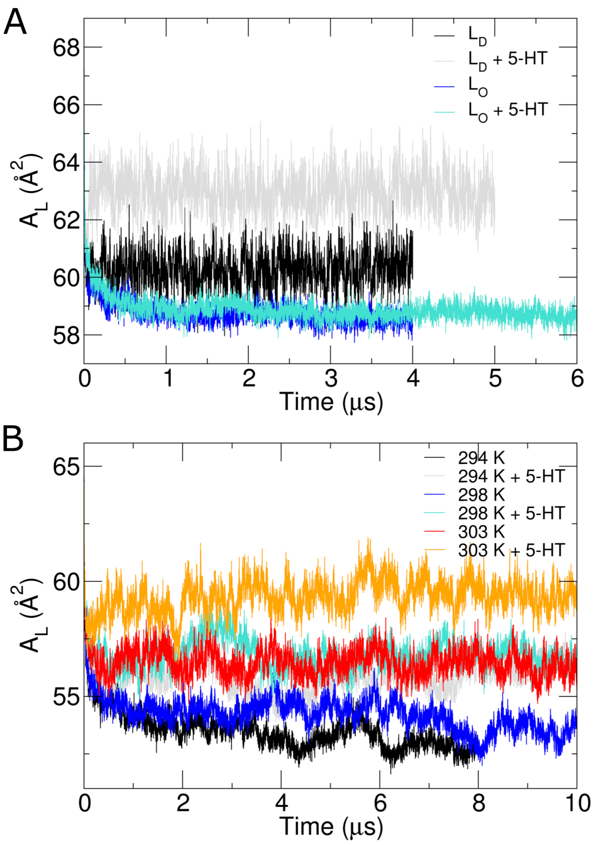

Figure S1. Local area per lipid. Local area per lipid as a function of time for the L_O and L_D systems (A) and all the $L_{O/D}$ systems (B).

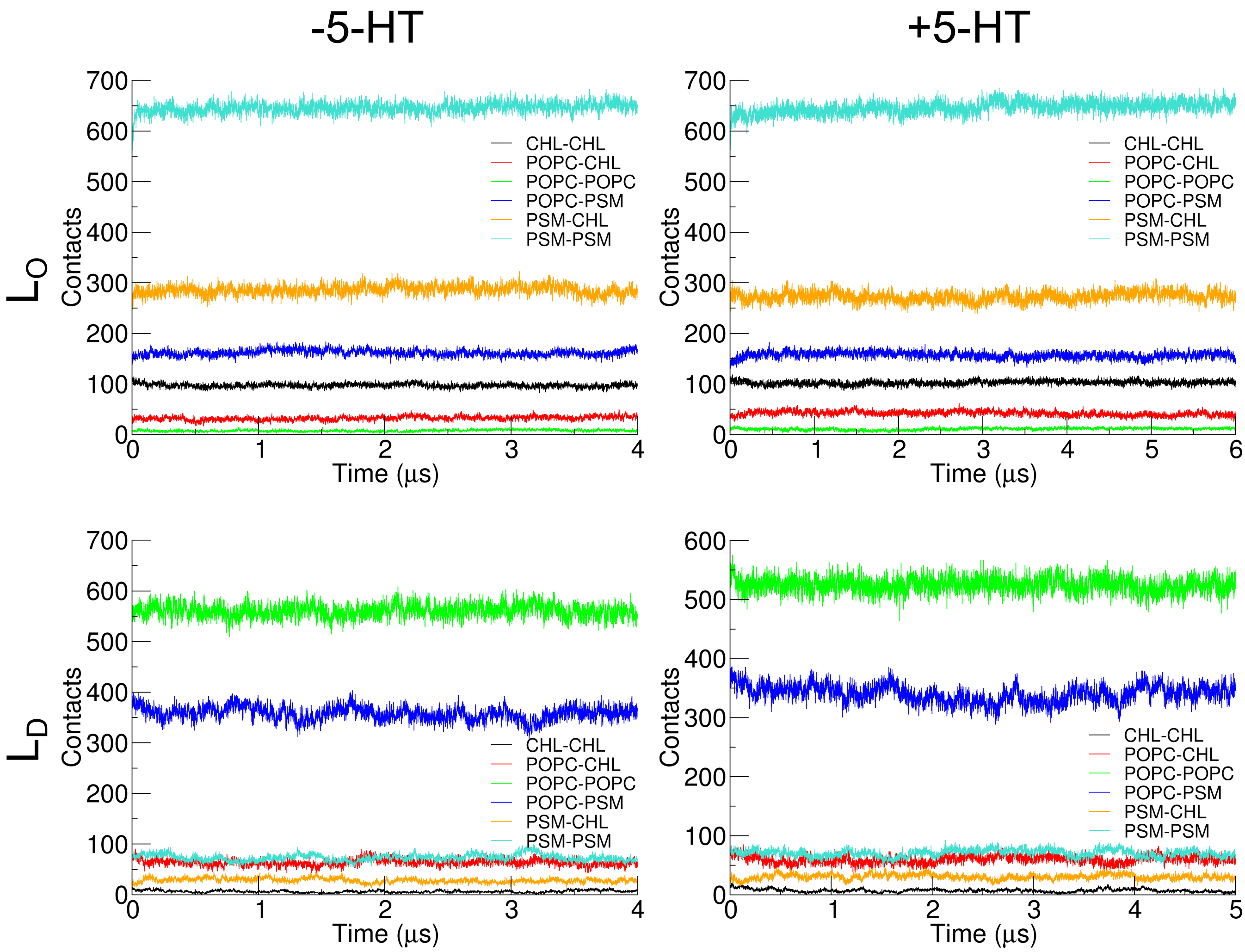

Figure S2. Contacts for the L_O and L_D systems. Contacts between each lipid type for the L_O and L_D systems with (+5-HT) and without (-5-HT) serotonin.

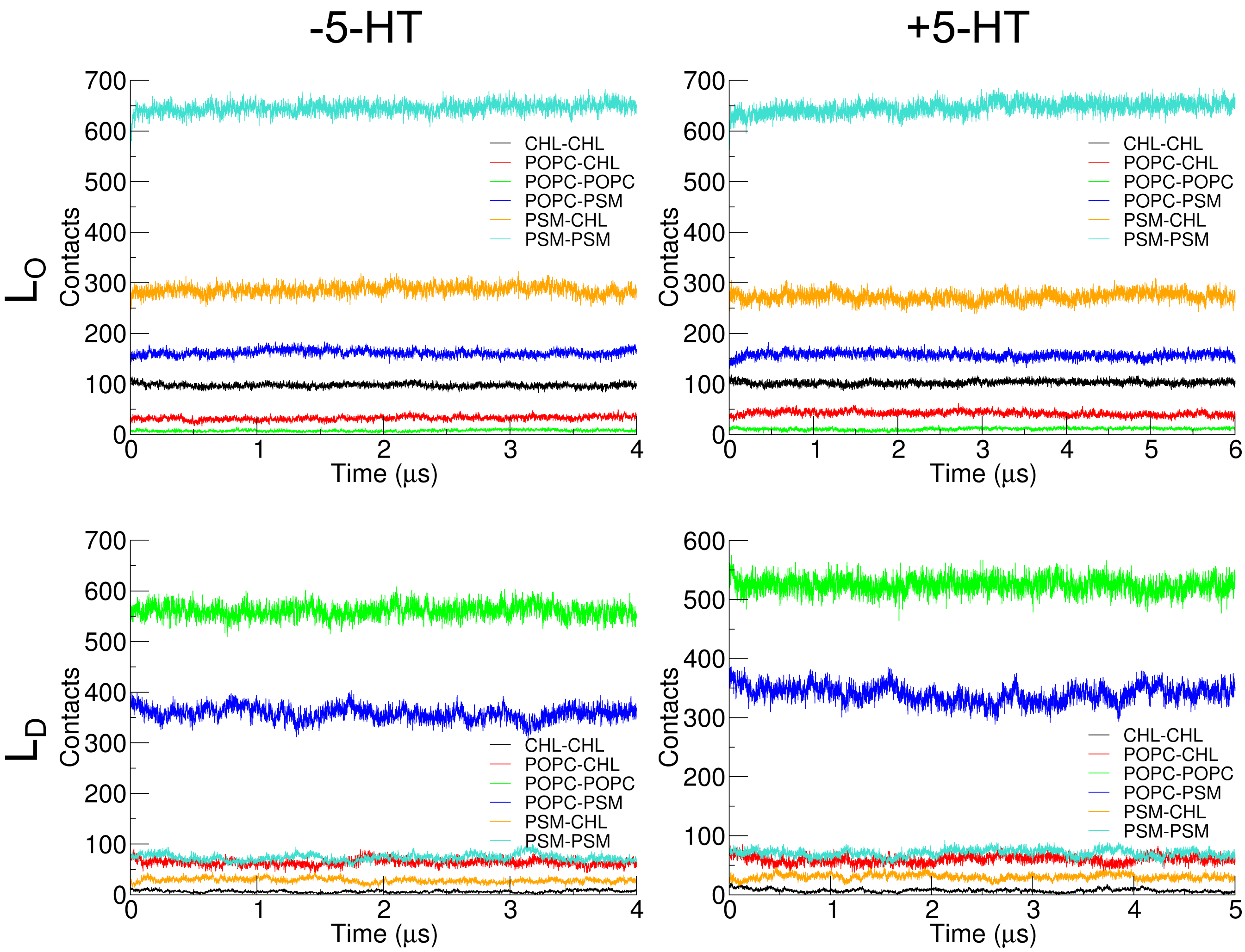

Figure S3. **Contacts for the $L_{O/D}$ systems.** Contacts are shown for the upper leaflet between the different lipid types for the LO/D systems at different temperatures with (+5-HT) or without (-5-HT) serotonin.

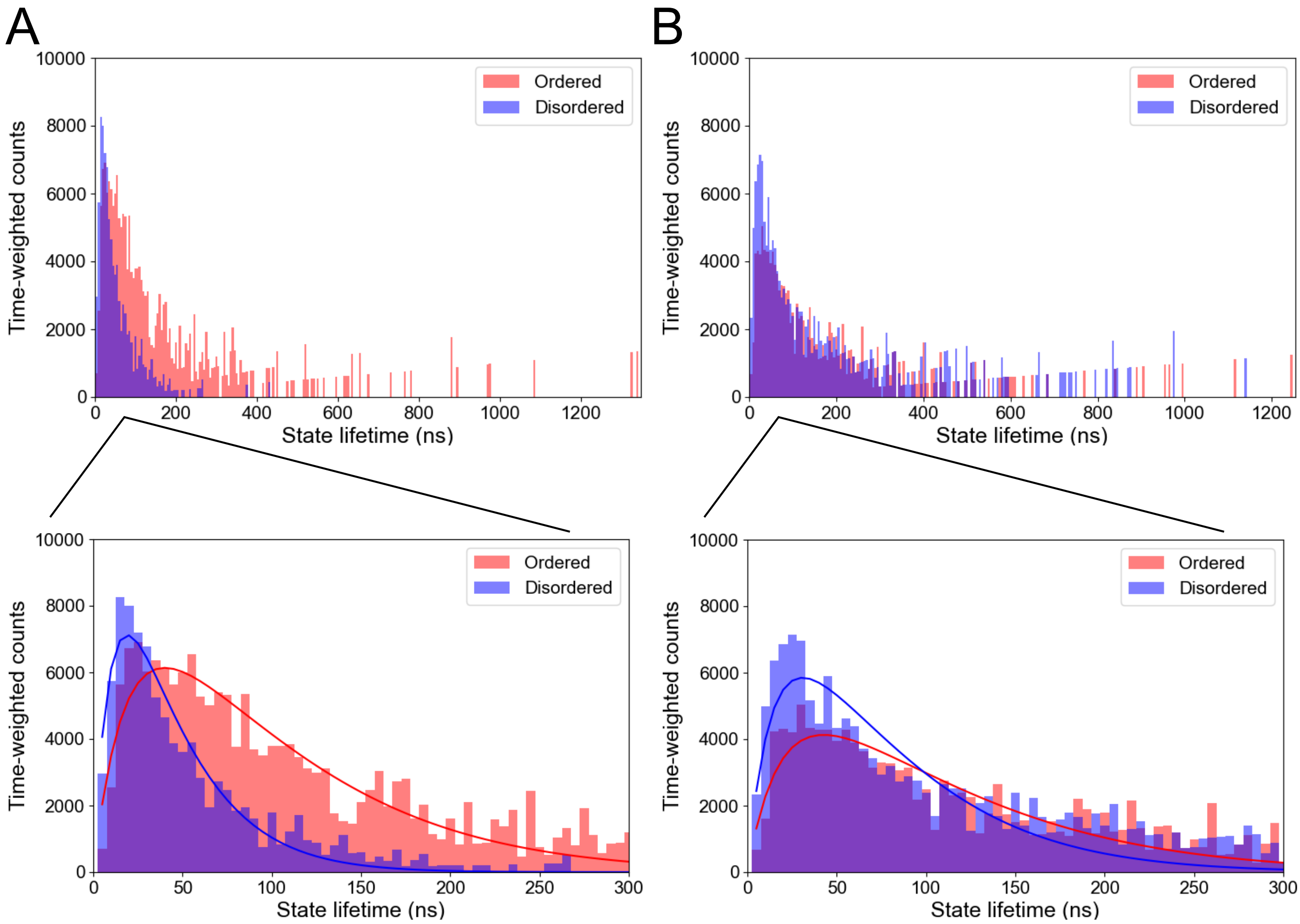

Figure S4. Time-weighted histograms of the hidden states lifetimes of POPC lipids for the $L_{O/D}$ system simulated at 303 K.. Column A and B correspond to the system without or with serotonin, respectively. The lines represent double exponential fits ($y = a_1 \cdot x \cdot e^{-x/\tau_1} + a_2 \cdot x \cdot e^{-x/\tau_2}$). The mean lifetimes ($\tau$) obtained for ordered POPC lipids are: 32 ns, 118 ns (w/o serotonin) and 30 ns, 130 ns (with serotonin). Analogously, for disordered POPC the values are: 17 ns, 51 ns (w/o serotonin) and 20 ns, 99 ns (with serotonin.

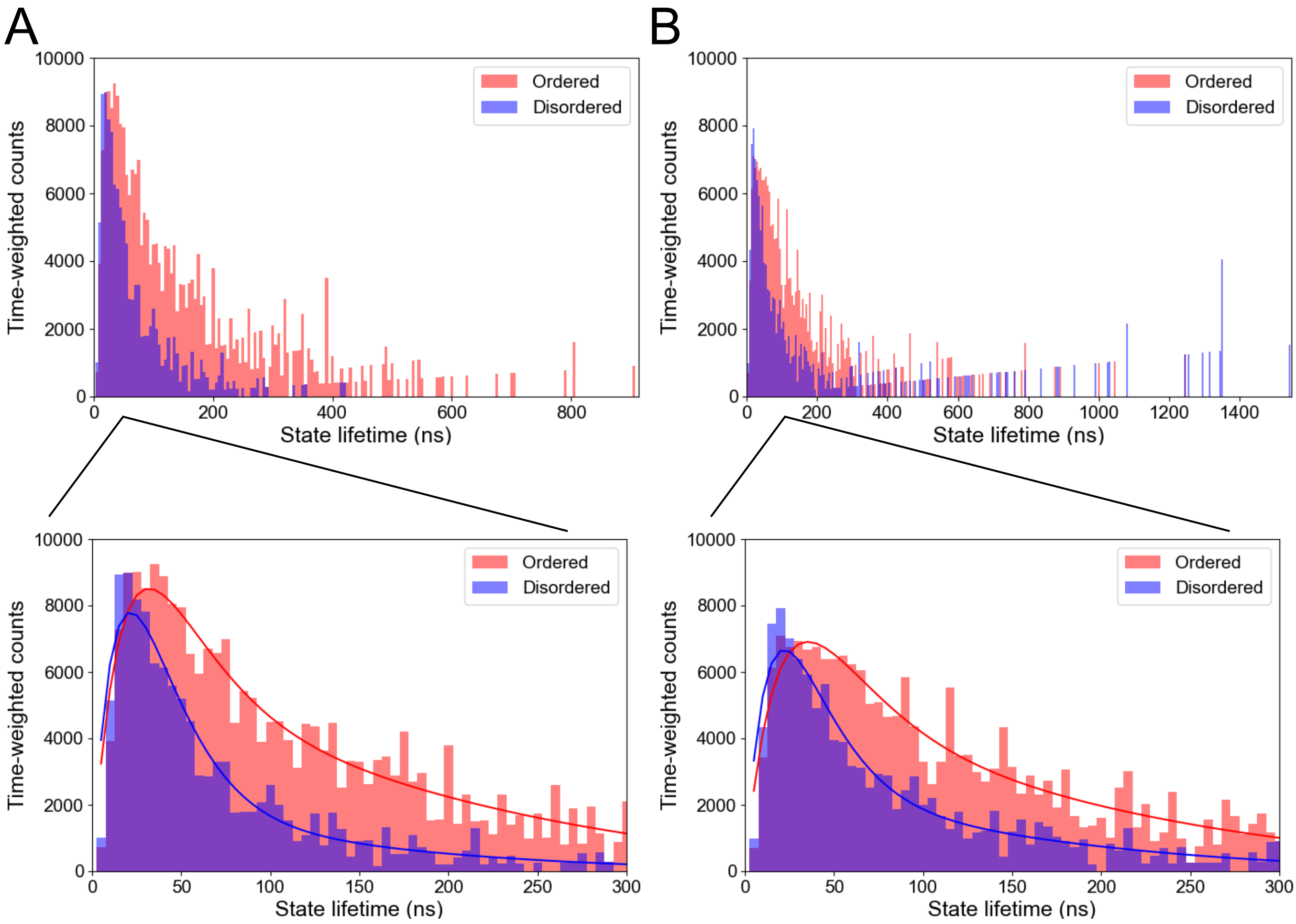

Figure S5. Time-weighted histograms of the hidden states lifetimes of PSM lipids for the $L_{O/D}$ system simulated at 303 K.. Column A and B correspond to the system without or with serotonin, respectively. The lines represent double exponential fits ($y = a_1 \cdot x \cdot e^{-x/\tau_1} + a_2 \cdot x \cdot e^{-x/\tau_2}$). The mean lifetimes ($\tau$) obtained for ordered PSM lipids are: 26 ns, 95 ns (w/o serotonin) and 29 ns, 97 ns (with serotonin). Analogously, for disordered PSM the values are: 20 ns, 76 ns (w/o serotonin) and 20 ns, 79 ns (with serotonin).
